# Root-secreted nucleosides: signaling chemoattractants of rhizosphere bacteria

**DOI:** 10.3389/fpls.2024.1388384

**Published:** 2024-05-10

**Authors:** Guy Keren, Galit Yehezkel, Lakkakula Satish, Zahar Adamov, Ze’ev Barak, Shimon Ben-Shabat, Varda Kagan-Zur, Yaron Sitrit

**Affiliations:** ^1^ The Jacob Blaustein Institute for Desert Research, Ben-Gurion University of the Negev, Beer-Sheva, Israel; ^2^ Department of Life Sciences, Ben-Gurion University of the Negev, Beer-Sheva, Israel; ^3^ Department of Clinical Biochemistry and Pharmacology, Faculty of Health Sciences, Ben-Gurion University of the Negev, Beer-Sheva, Israel; ^4^ Katif Research Center for Research & Development, Netivot, Israel

**Keywords:** chemotaxis, chemoattractants, rhizobacteria, nucleosides, root exudates

## Abstract

The rhizosphere is a complex ecosystem, consisting of a narrow soil zone influenced by plant roots and inhabited by soil-borne microorganisms. Plants actively shape the rhizosphere microbiome through root exudates. Some metabolites are signaling molecules specifically functioning as chemoattractants rather than nutrients. These elusive signaling molecules have been sought for several decades, and yet little progress has been made. Root-secreted nucleosides and deoxynucleosides were detected in exudates of various plants by targeted ultra-performance liquid chromatography–mass spectrometry/mass spectrometry. Rhizobacteria were isolated from the roots of *Helianthemum sessiliflorum* carrying the mycorrhizal desert truffle *Terfezia boudieri*. Chemotaxis was determined by a glass capillary assay or plate assays on semisolid agar and through a soil plate assay. Nucleosides were identified in root exudates of plants that inhabit diverse ecological niches. Nucleosides induced positive chemotaxis in plant beneficial bacteria *Bacillus pumilus*, *Bacillus subtilis*, *Pseudomonas turukhanskensis* spp., *Serratia marcescens*, and the pathogenic rhizobacterium *Xanthomonas campestris* and *E coli*. In a soil plate assay, nucleosides diffused to substantial distances and evoked chemotaxis under conditions as close as possible to natural environments. This study implies that root-secreted nucleosides are involved in the assembly of the rhizosphere bacterial community by inducing chemotaxis toward plant roots. In animals, nucleoside secretion known as “purinergic signaling” is involved in communication between cells, physiological processes, diseases, phagocytic cell migration, and bacterial activity. The coliform bacterium *E. coli* that inhabits the lower intestine of warm-blooded organisms also attracted to nucleosides, implying that nucleosides may serve as a common signal for bacterial species inhabiting distinct habitats. Taken together, all these may indicate that chemotaxis signaling by nucleosides is a conserved universal mechanism that encompasses living kingdoms and environments and should be given further attention in plant rhizosphere microbiome research.

## 1 Introduction

Many ecologically important symbiotic and mutually beneficial relationships rely on host plants’ recruitment of microbial partners from the environment. One mechanism used by bacteria to locate a potential host is chemotaxis, i.e., direct active movement toward or away from chemical signals (Raina et al., 2019). Chemotaxis toward plant roots may be initiated by two driving forces: nutritional signals and specific chemoattractants, including volatiles (Schulz-Bohm et al., 2018). Such specific signals affect the chemotactic behavior of plant rhizobacteria, particularly those that induce host–plant structural remodeling (Chepsergon and Moleleki, 2023). Chemotactic responses to root exudates are initiated by ligand sensing at chemoreceptors or methyl-accepting chemotaxis proteins (MCPs). Many MCPs and the corresponding chemoeffectors were identified in soil plant growth–promoting bacteria (PGPBs) (Feng et al., 2021). Plants must apply mechanisms to restrict the association with pathogenic bacteria (Matilla and Krell, 2018); however, recognition signals are occasionally common between beneficial and pathogenic bacteria, such as in the case of beneficial rhizobia species and the causative agent of crown gall disease *Rhizobium radiobacter* (Matilla and Krell, 2018). A fascinating example that demonstrates the evolution of such signaling chemoattractants, throughout several kingdoms, in both beneficial and pathogenic associations, is strigolactones, which are involved in plant–parasite interactions of *Striga*, *Orobanche*, *Alectra*, *Phelipanche* and plant–bacteria/fungal interactions including endophytes and endomycorrhiza (Akiyama et al., 2005; Tsai et al., 2020). The array of root-secreted compounds is immense, even in the exudate of a single plant species (Strehmel et al., 2014; Tawaraya et al., 2014; Warren, 2015; Tang et al., 2022), the composition of which varies according to the species, developmental stage, environmental conditions, microbiome, and even along the root zones (Canarini et al., 2019). In turn, temporal and spatial variations in bacterial behavior are common in the rhizosphere (Massalha et al., 2017). Nucleosides have been identified as root-secreted compounds in exudates of a few plant species (Strehmel et al., 2014; Tawaraya et al., 2014; Warren, 2015; Tang et al., 2022). However, recent works, using metabolomic approach, discovered that nucleoside secretion from roots is a common phenomenon of plants (McLaughlin et al., 2023) and that profound changes in their contents occur during plant development (Delgado-García et al., 2021). Nucleosides are mainly utilized as nutrients when other carbon sources such as sugars and amino acids are exhausted. Specific MCPs for nucleosides have not been characterized in rhizobacteria, and the identified purine binding chemoreceptor McpH of *Pseudomonas putida* KT2440 does not bind nucleosides (Fernández et al., 2016). The search for chemoreceptor ligand binding domains by Pfam 31.0 and UniProt reference proteome databases revealed only one putative nucleoside binding domain, the gate domain model, and only few nucleotides binding domains (Ortega et al., 2017). Being building blocks of nucleic acids, nucleoside secretion is energetically and nutritionally demanding especially in early development of plants (Delgado-García et al., 2021), implying that they may act as signaling molecules rather than as nutrients. Consequently, we hypothesized that root-secreted nucleosides serve as signaling molecules that play a key role in the assemblage and configuration of the rhizosphere microbiome. We thus set out to study nucleoside exudation in diverse plant species inhabiting various ecozones, from desert to tropical, monocots and dicots, mycorrhizal and non-mycorrhizal, and plants forming bacterial symbioses. These included *Helianthemum sessiliflorum* (Desf.) Pers that forms ecto/endo-mycorrhiza with desert truffles (Turgeman et al., 2011); *Arabidopsis thaliana* Col0 known not to form mycorrhiza; *Zea mays* (L.) (maize) representing monocots; *Vigna radiata* (L.) Wilczek (mung-bean) and *Lens culinaris* ssp. *culinaris* (lentil), both legumes that form symbiosis with *Rhizobia* and endo-mycorrhiza; and *Persea americana* Mill. (avocado), a tree originating in tropical forests. Finally, we investigated whether such nucleosides could evoke chemotactic response in various bacteria.

## 2 Materials and methods

### 2.1 Plant culture

Six plant species originating from different ecosystems were chosen for this study: *Helianthemum sessiliflorum*, the desert shrub host of the desert truffle *Terfezia boudieri*; *Arabidopsis thaliana* Col0, representing non-mycorrhizal plants; *Vigna radiata* (mung beans) and *Lens culinaris* (lentil), legumes that form a chemo-communication system with nitrogen-fixing bacteria (Rhizobia) and endomycorrhizal fungi; *Zea mays* (maize), representing monocotyledons; and *Persea americana* (avocado), representing a tree originating in tropical forests. Seeds of *V. radiata*, *L. culinaris*, maize, and avocado were purchased at a local market, and seeds of *H. sessiliflorum* were collected in April 2018 from the experimental plot at the Bergman campus, Ben-Gurion University of the Negev, Beer Sheva.

Plants were cultured under sterile, controlled conditions for analysis of polar and semi-polar compounds in root exudates as follows: seeds were surface-disinfected by immersion in 70% ethanol, rinsed three times with double distilled water (DDW), immersed in 1% NaOCl (containing a drop of Tween 20), and rinsed again with DDW. For *H. sessiliflorum* and *P. americana*, the seed coat was removed by gentle scraping with sandpaper prior to surface disinfection. *H. sessiliflorum* seeds were soaked in sterile DDW at 6°C for at least 24 h before sowing. Seeds were sown manually in Magenta GA-7 Plant Culture Boxes (bioWORLD) with sterile Perlite (particle size, 4; 78% under 2.4–4.8 mm/22% under 2.4 mm, Agrical) except for *A. thaliana* seeds that were mixed with 0.4% agar in the same medium. The growth medium was Murashige and Skoog (MS)-zero (Murashige and Skoog, 1962), named N5 containing (in mg/L): 0.025, CoCl_2_; 0.025, CuSO_4_; 36.7, Fe EDTA; 6.2, H_3_BO_3_; 0.83, KI; 16.9, MuSO_4_; 0.25, Na_2_MoO_4_; 8.6, ZnSO_4_; 332.02, CaCl_2_; 170, KH_2_PO_4_; 380, KNO_3_; 180.54, MgSO_4_; 330, NH_4_NO_3_; 0.5, pyridoxine; 2.0, glycine; 100, myo-inositol; 0.1, thiamine HCl; and 0.5, nicotinic acid (pH 5.8; adjusted with 10% KOH). Avocado was grown with sterile DDW. The boxes were placed for 44 days in a controlled growth room under 24 ± 2°C, long daylight (16 h), and fluorescent light (TL-D 36W/54-765, cool daylight). Control magenta boxes contained the matrix without seeds.

### 2.2 Extraction of root exudates

Root exudates were collected by sequential immersion of the perlite in DDW. The perlite was removed by vacuum filtration on the filter paper (Whatman®, No. 1). The combined filtered solution was lyophilized, and the dry powder was dissolved in DDW to a concentration of 100 mg/mL and filtered through 0.22-μm filter prior to chemical and physical analysis.

### 2.3 Chemical characterization of root exudates

The root exudate was analyzed in the presence of nucleosides and deoxynucleosides using ultra-performance liquid chromatography–mass spectrometry/mass spectrometry (UPLC-MS/MS). A mixture of nine synthetic nucleosides served as the standard.

### 2.4 UPLC-MS/MS system

The system included an ACQUITY UPLC® I Class Waters system with a Q Exactive, Thermo Scientific MS detector (Electrospray Ionization) equipped with ACQUITY UPLC®HSS T3 1.8-μm (2.1 mm × 100 mm) column. The mobile phase A was 0.1% formic acid (FA) in DDW (ULC/MS), and mobile phase B was 0.1% FA in acetonitrile (ULC/MS). Gradient sequence was as follows: 0 min to 1 min, 99%–95% A and 1%–5% B; 1 min to 8 min, 95%–1% A and 5%–99% B; 8 min to 10.9 min, isocratic 1% A and 99% B; 10.9 min to 11 min, 1%–5% A and 99%–95% B; 11 min to 13 min, 5%–99% A and 95%–1% B; and 13 min to 15 min, isocratic 99% A and 1% B, at a flow rate of 0.4 mL/min. The injection volume was 5 μL, and the column and sample tray temperatures were 40°C and 10°C, respectively. MS scan range was 100–1,500 m/z in both positive and negative modes, and data were analyzed using Thermo Xcalibur™ 4.0 software (Thermo Fisher Scientific). Identification of the synthetic standards’ mass to charge ratio (m/z) was done by calculating their theoretical m/z value, identifying the specific m/z’s retention time (RT), and comparing the m/z readings of both individual standard solutions and mixed solution ( ± 0.001) and comparing to Thermo Fisher Scientific mzCloud Mass Spectral Library™. In addition, extracted ion chromatograms were generated for each compound in both the standard and sample solutions to compare the peak profile as another layer of confidence.

### 2.5 Bacterial culture

Bacteria including *B. subtilis* [strain 168, American Type Culture Collection (ATCC)], a motile model PGPB (Wadhams and Armitage, 2004; Pérez-García et al., 2011) *B. pumilus* b-13, *Serratia marcescens*, and *Pseudomonas turukhanskensis* were isolated from the rhizosphere of *H. sessiliflorum*. *Xanthomonas campestris* served as a model plant pathogen. Soil bacteria were stored in 25% glycerol (v/v) stock at −75°C and grown in Luria broth (LB) at 30°C. *Escherichia coli* K-12 MG1655 (that approximates wild-type *E. coli*) was employed in chemotaxis assay as a common/general strain. The *E. coli* was grown in Tryptone broth (TB) at 34°C for chemotaxis in-plug assay. To determine *E. coli* and *B. pumilus* b-13 growth when the carbon source was nucleoside, bacteria were grown in M9 minimal media supplemented with 1 mM 2-deoxy adenosine (2DA) substituting the glucose.

### 2.6 Capillary chemotaxis assay

Bacterial colony was grown overnight in LB, at 30°C in a rotary shaker (250 rpm). The cell suspension was diluted 1:100 to LB medium and was grown; for *Bacilli* species, until it reached O.D. (O.D.)_600nm_ of 0.5–0.6; and for *X. campestris*, until optical density_600nm_ of 0.9–1.2. Bacteria motility was verified by a phase microscope (BH-2, OLYMPUS). These O.D. points were determined due to the highest observed motility of each strain. A solution of 5% glycerol and 0.5 M Na + lactate was added to a final volume of 1%, and the culture was incubated for 15 min with an occasional mixing. Cells were washed twice (centrifuged 3,200xg, 3 min, 25°C) and replaced with chemotaxis buffer (CB) containing 10 mM K_3_PO_4_ (pH 6.7), 0.14 mM CaCl_2_, 5 mM Na + lactate, 0.3 mM (NH_4_)_2_SO_4_, 0.05% (v/v) glycerol, and 0.1 mM EDTA (pH 7). For *X. campestris*, 1 mM MgCl_2_ was added to the CB. The bacterial cultures were resuspended, centrifuged, and washed twice. The bacterial suspension was diluted in CB buffer to an O.D._600nm_ of 0.005. Chemotaxis was determined by a glass capillary assay (Adler, 1973). The assay included solutions of nucleosides in CB, positive control of amino acids (1 mM asparagine for *B. subtilis* 168 and *B. pumilus* b-13, and 5 mM methionine for *X. campestris*), and negative control (CB), in which methionine served as a positive control based on a previous study with *Xanthomonas oryzae* (Kumar Verma et al., 2018). A 60-μL solution was loaded into three one-end sealed capillary tube (100 mm × 1.2 mm, Marienfeld Laboratory Glassware, Lauda-Königshofen, Germany) under vacuum. Capillaries were positioned vertically in a 96-well plate, each well contained 270 μL of bacterial suspension and incubated for 30 min at 30°C. Then, each capillary was removed, externally washed with DDW, and broken into an Eppendorf tube to discharge the solution. A sample of 40 μL was serially diluted and from each dilution, and 100 μL was mixed with 3.5 mL of Top agar (0.8% agar, 0.8% NaCl, and 1% tryptone) and plated. The plates were incubated overnight at 30°C, and colonies were counted. The results are presented as colony-forming units (CFU) per capillary.

### 2.7 In-plug chemotaxis assay

*E. coli*, *S. marcescens*, and *P. turukhanskensis* colonies were grown overnight in TB (1% Tryptone and 0.5% NaCl) at 34°C. Then, the cell suspension was diluted with TB and re-grown to O.D._600nm_ of 1 to obtain vigorous motility. Cells were precipitated (4,000 rpm, 6 min, 25°C) and washed with a solution of 10 mM K_3_PO_4_ (pH 7.2), 0.1 mM EDTA, and 0.001 mM methionine. The cell pellet was resuspended with 10 mL of the same buffer and mixed 1:1 with a buffer containing 0.8% agar and poured into plates containing three plugs of 2% agar, with or without 0.1 mM or 1 mM 2DG. The plates were allowed to solidify and then incubated at 30°C for 2 h to 4 h. Following incubation, the plates were inspected for turbid growth around the agar plugs and a circling clear zone, indicating bacterial chemotaxis toward the plug containing nucleosides.

### 2.8 Soil chemotaxis assay

Chemoattraction of *B. pumilus* b-13 to 2-deoxy-guanosine (2DG) in soil was tested in a plate assay [adapted from Paul et al. (2006), with major adjustments]. *B. pumilus* b-13 was grown overnight in LB, and the bacterial culture was diluted and re-grown from an O.D._600nm_ of 0.05 to ~0.8 (when bacterial motility was vigorous). The plates were prepared as follows: A solidifying medium with 6.6 mM MgSO_4_, 6.05 mM (NH_4_)_2_SO_4_, yeast extract (0.02 mg/mL), and 0.4% agarose in 110 mL of phosphate buffered saline (PBS), with or without 0.1 mM 2DG, was poured into 145-mm-diameter Petri dish. An inner circle of the agarose medium was cut out to leave an outer ring of 1-cm width. The plate was filled with sterilized dune sand (taken from the rhizosphere from which b-13 was isolated) moistened to 20% w/w with PBS. A bacterial sample of 10^9^ cells/0.1 mL was dripped onto a shallow pit in the center of the plate that was next incubated at 30°C, overnight. The following morning, sand samples of ~130 mg were taken from four spots at the perimeter of the sand layer (a radius of ~6 cm) of each plate and combined. Each sand sample was suspended in 1 mL of PBS and serially diluted, and an aliquot of 0.1 mL was plated for CFU counting. The results were normalized to soil weight.

### 2.9 Statistical analysis

All experiments were repeated twice or more, and chemotaxis experiments were carried with triplicate capillaries per treatment. Significant differences were calculated by one-way ANOVA, followed by Tukey’s range test (α ≤ 0.05).

## 3 Results

### 3.1 Nucleosides are exuded by roots of various plant species

Root-secreted nucleosides and deoxynucleosides were detected in all tested plants by targeted UPLC-MS/MS (**Table 1**). Uridine, adenosine, and 2-deoxy-adenosine (2DA) were ubiquitous in all plants, whereas 2DG and thymidine were identified by MS2 in all samples except those from *P. americana* (**Table 1**). These results correspond with previous reports for *A. thaliana* (Strehmel et al., 2014), monocotyledons (*Triticum aestivum* L.) (Warren, 2015), and the legume *Phaseolus vulgaris* L (Tawaraya et al., 2014). However, guanosine was not detected in *A. thaliana* as previously shown (Strehmel et al., 2014). In some samples, several nucleoside fragments were not detected by MS2, possibly due to low amounts that were below the detection threshold. Cytidine fragmentation occurred only in legume extracts, indicating its high secretion rate in these species (**Table 1**). The relative abundance of 2-deoxy-cytidine was very low, as it was detected only in the MS1 (m/z, 228.0973 ± 0.001) of all samples except *P. americana*. These results suggest that nucleoside secretion is indeed a common phenomenon among plants.

**Table 1:** Nucleoside and deoxynucleoside composition of root exudates.

			*Helianthemum sessiliflorum*				*Arabidopsis thaliana* Col-0				*Zea mays* (maize)			
Nucleoside	Elemental composition	Type of ionization	RT (min)	MS1	MS2	Verification	RT (min)	MS1	MS2	Verification	RT (min)	MS1	MS2	Verification
				m/z	Observed fragments, precursor ion in bold (m/z)			m/z	Observed fragments, precursor ion in bold (m/z)			m/z	Observed fragments, precursor ion in bold (m/z)	
Cytidine	C9H13N3O5	(M+H)+	0.86	244.092	–	MS1	0.87	244.092	–	MS1	0.84	244.092	–	MS1
2-Deoxy-Cytidine	C9H13N3O4	(M+H)+	0.86	228.097	–	MS1	0.87	228.097	–	MS1	0.86	228.097	–	MS1
Uridine	C9H12N2O6	(M-H)-	1.48	243.063	**243**, 200, 110, 152, 82	MS1,MS2	1.47	243.063	**243**, 200, 152, 110, 82, 66	MS1,MS2	1.47	243.063	**243**, 200, 152, 110, 82, 66	MS1,MS2
		(M+FA-H)		289.068				289.068				289.068		
Adenosine	C10H13N5O4	(M+H)+	1.69	268.104	**268**, 136	MS1, MS2	1.69	268.104	**268**, 136	MS1,MS2	1.7	268.104	**268**, 136	MS1,MS2
2-Deoxy-Adenosine	C10H13N5O3	(M+H)+	1.75	252.109	**252**, 136, 117	MS1, MS2	1.76	252.109	**252**, 136, 117	MS1,MS2	1.75	252.109	**252**, 136, 117	MS1,MS2
Guanosine	C10H13N5O5	(M-H)-	1.79	282.085	**282**, 150, 133	MS1, MS2	1.79	282.085	–	MS1	1.78	282.085	**282**, 150, 133	MS1,MS2
		(M+FA-H)		328.09				328.09				328.091		
2-Deoxy-Guanosine	C10H13N5O4	(M+H)+	1.88	268.104	**268**, 152, 117	MS1, MS2	1.89	268.104	**268**, 152, 117	MS1,MS2	1.9	268.104	268, 152, 117	MS1,MS2
Thymidine	C10H14N2O5	(M-H)-	2.2	241.084	**241**, 151,125	MS1, MS2	2.2	241.083	**241**, 151,125	MS1,MS2	2.21	241.083	**241**, 151,125	MS1,MS2
		(M+FA-H)		287.09				287.089				287.089		
			*Vigna radiata* (mung bean)				*Lens culinaris* (lentil)				*Persea americana* (avocado)			
Nucleoside	Elemental composition	Type of ionization	RT (min)	MS1	MS2	Verification	RT (min)	MS1	MS2	Verification	RT (min)	MS1	MS2	Verification
				m/z	Observed fragments, precursor ion in bold (m/z)			m/z	Observed fragments, precursor ion in bold (m/z)			m/z	Observed fragments, precursor ion in bold (m/z)	
Cytidine	C9H13N3O5	(M+H)+	0.84	244.092	**244**, 95, 112	MS1,MS2	0.86	244.092	**244**, 95, 112	MS1,MS2	0.86	244.092	–	MS1
2-Deoxy-Cytidine	C9H13N3O4	(M+H)+	0.86	228.097	–	MS1	0.87	228.097	–	MS1	0.87	–	–	MS1
Uridine	C9H12N2O6	(M-H)-	1.48	243.063	**243**, 200, 152, 110, 82, 66	MS1,MS2	1.47	243.063	**243**, 200, 152, 110, 82, 66	MS1,MS2	1.49	243.063	**243**, 200, 152, 110, 82, 66	MS1,MS2
		(M+FA-H)		289.069				289.069				289.069		
Adenosine	C10H13N5O4	(M+H)+	1.71	268.104	**268**, 136	MS1,MS2	1.69	268.103	**268**, 136	MS1,MS2	1.71	268.103	**268**, 136	MS1,MS2
2-Deoxy-Adenosine	C10H13N5O3	(M+H)+	1.76	252.109	**252**, 136, 117	MS1,MS2	1.77	252.109	**252**, 136, 117	MS1,MS2	1.79	252.109	**252**, 136, 117	MS1,MS2
Guanosine	C10H13N5O5	(M-H)-	1.77	282.085	**282**, 150, 133	MS1,MS2	1.79	282.085	**282**, 150, 133	MS1,MS2	1.8	282.085	–	MS1
		(M+FA-H)		328.09				328.09				–		
2-Deoxy-Guanosine	C10H13N5O4	(M+H)+	1.92	268.104	268, 152, 117	MS1,MS2	1.86	268.104	**268**, 152, 117	MS1,MS2	1.92	268.104	–	MS1
Thymidine	C10H14N2O5	(M-H)-	2.21	241.083	**241**, 151,125	MS1,MS2	2.2	241.083	**241**, 151,125	MS1,MS2	2.2	241.084	–	MS1
		(M+FA-H)		287.089				287.089				287.089		

Compound identification and verification by UPLC-MS/MS analysis were based on retention time (RT), mass spectrum, and chromatographic profile compared to authentic standards. In the MS2, precursor ions are marked in bold.

### 3.2 Nucleosides induce chemotaxis in soil-borne bacteria

**Figure 1:**
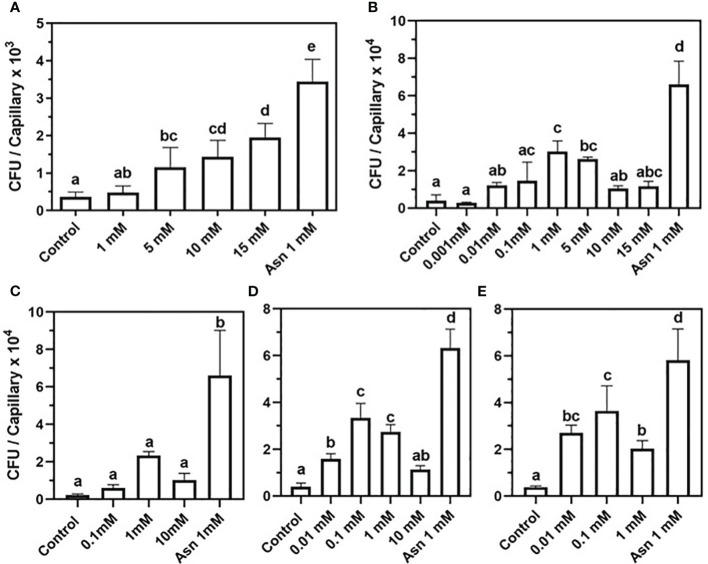
Chemotaxis of *Bacillus subtilis* 168 and *Bacillus pumilus* b-13 toward nucleosides in capillary assay. *B*. *subtilis* 168: **(A)** 2-deoxy-guanosine. *B*. *pumilus* b-13: **(B)** 2-deoxy-guanosine, **(C)**- thymidine, **(D)** 2-deoxy-adenosine, and **(E)** guanosine. The bacterial suspension was grown to an early exponential phase and suspended in chemotaxis buffer (CB). Chemoattraction was determined by the number of colony-forming units (CFU) in capillaries (n = 3). Negative control was CB, and positive control was 1 mM asparagine (Asn 1 mM). Data are means ± SD, N = 2–3. Lowercase letters denote statistical significance (α ≤ 0.05) according to one way-ANOVA followed by Tukey’s Honest Significant Difference test (HSD).

To test whether nucleosides induce chemotaxis in soil-borne bacteria, a capillary assay was performed. The model soil bacteria were *Bacillus subtilis* 168 and *Bacillus pumilus*, which are PGPB, and *Xanthomonas campestris*, which is a generalist plant pathogen. The *B. pumilus* strain, termed b-13, was isolated from the rhizosphere of mycorrhized *H. sessiliflorum* plants. Both PGPB *B. subtilis* 168 and *B. pumilus* b-13 exhibited a positive chemotaxis toward nucleosides yet with different sensitivities and response trends. A positive correlation was observed between the amount of *B. subtilis* 168 cells accumulated within capillaries (expressed by CFU) and 2DG concentrations, up to the highest level of 15 mM (**Figure 1A**). The CFU values were significantly different between capillaries loaded with 5 mM, 10 mM, and 15 mM of 2DG with 1,160 CFU, 1,430 CFU, and 1,950 CFU per capillary, respectively. A non-significant difference between the CFU values of the control, with CB alone, and 1 mM 2DG implies that the threshold level for attraction ranges between 1 mM and 5 mM 2DG. Control capillaries showed the lowest number of cells with 363 CFU per capillary, whereas the CFU value of the positive control, 1 mM asparagine, was the highest (3,440 CFU per capillary).

To further verify that secreted nucleosides are indeed potent chemoattractants of soil bacteria, their effect on *B. pumilus* b-13 was tested. A bell-shaped chemotaxis response was observed for all nucleosides, yet its magnitude varied between compounds (**Figures 1B–E**). For 2DG, chemotaxis peaked at 1 mM with nine-fold higher colony counts than the control with 30,190 CFU and 3,933 CFU per capillary, respectively (**Figure 1A**). The higher and lower concentrations of 2DG (15 mM, 10 mM, 0.01 mM, and 0.001 mM) also induced chemotaxis, but to a lesser extent (11,680 CFU, 10,530 CFU, 12,180 CFU, and 2,800 CFU per capillary, respectively). Thus, the response of *B. pumilus* toward 2DG saturated between 0.1 mM and 5 mM. This bell-shaped response of *B. pumilus* b-13 differs from the concentration-depended, almost linear, response of *B. subtilis* 168. Additionally, *B. pumilus* b-13 CFU values were nine-fold higher compared to those of *B. subtilis* 168, indicating that the former has superior motility.

Similar to 2DG, thymidine induced a bell-shaped chemotactic response in *B. pumilus* (**Figure 1B**), peaking at 1 mM with cell counts 10-fold higher than the control (23,370 CFU and 2,313 CFU per capillary for both, respectively). Lower or higher thymidine concentrations of 10 mM and 0.1 mM yielded 10,190 CFU and 6,608 CFU per capillary, whereas the lowest levels of 0.01 mM and 0.001 mM were similar to controls (data not shown). In assays employing 2DA and guanosine (**Figures 1D, E**), the chemoattraction was observed at 0.1 mM with nine-fold higher colony counts compared to the control for both nucleosides (33,400 and 3,943 for 2DA, and 36,400 and 3,824 CFU per capillary for guanosine). Insignificant reduction in colony counts was observed for 1 mM 2DA (27,350 CFU per capillary) and 0.01 mM guanosine (27,060 CFU per capillary), indicating that maximal chemoattraction occurs between 0.1 mM and 1 mM for 2DA and between 0.01 mM and 0.1 mM for guanosine. A comparison between *B. pumilus*’ response to various nucleosides showed that, while 2DG and thymidine evoked maximal chemotaxis at 1 mM, its sensitivity to 2DA and guanosine was higher, with a response already peaking at 0.1 mM.

**Figure 2:**
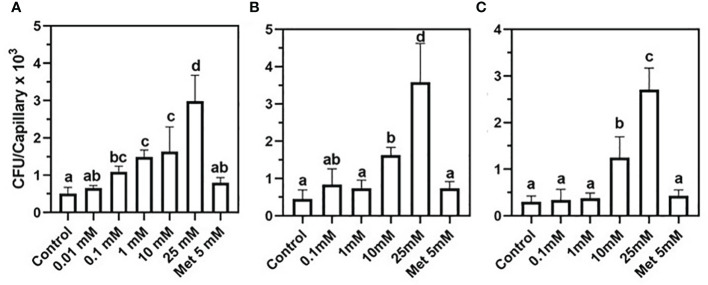
Chemotactic of the plant pathogen *Xanthomonas campestris* toward nucleosides. **(A)** 2-Deoxy-guanosine, **(B)** 2-deoxy-adenosine, and **(C)** thymidine. Positive control was 5 mM methionine (Met 5mM). Bacteria were grown to a late exponential phase and suspended in chemotaxis buffer (CB). Chemoattraction was determined by the number of colony-forming units (CFU) (n = 3). Data are means ± SD, N = 3. Lowercase letters denote significance between treatments calculated according to Tukey’s multiple comparisons test (α ≤ 0.05).

To test whether phytopathogens can exploit nucleoside signaling as well, *Xanthomonas campestris*, a Gram-negative, motile generalist pathogen was chosen (Ryan et al., 2011). A positive correlation was discovered between nucleoside concentrations and the number of attracted *X. campestris* cells (**Figures 2A–C**). The data in **Figure 2A** demonstrate a positive chemotactic response of *X. campestris* toward 2DG although to a lesser extent than that of *B. pumilus* b-13 (**Figure 1B**). The most robust chemotactic response was observed at 25 mM 2DG, with an average of 2,986 CFU per capillary (**Figure 2A**). Significant chemotaxis occurred at the lower 2DG concentrations of 0.1 mM, 1 mM, and 10 mM, with 1,095 CFU, 1,494 CFU, and 1,630 CFU per capillary, compared to the control (507 CFU per capillary). Methionine that served as a positive control based on a previous study with *Xanthomonas oryzae* (Kumar Verma et al., 2018) showed slightly higher (136%) insignificant chemotaxis compared to the control (796 CFU per capillary).

A similar trend of positive correlation between chemotactic response and nucleoside concentration was observed with 2DA and thymidine (**Figures 2B, C**). For both nucleosides, chemotaxis peaked at 25 mM with 3,585 CFU and 2,706 CFU per capillary, whereas the control CFU counts were lower by 88%. Lower nucleoside concentrations and the methionine positive control showed 42%–62% more CFUs compared to CB controls. The presented data demonstrate that nucleosides induce positive chemotaxis in both pathogenic and PGPB, although the sensitivity of *X. campestris* was significantly lower compared *to B. pumilus* b-13.

### 3.3 2-Deoxy-adenosine induces chemotaxis in warm-blooded associated bacteria

**Figure 3:**
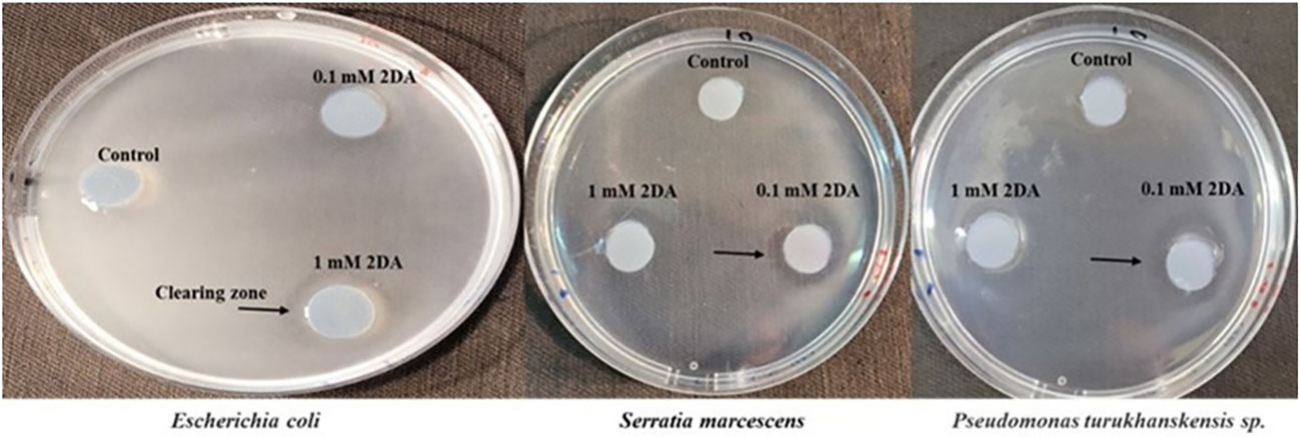
Chemotaxis plate assay of *Escherichia coli*, *Serratia marcescens*, and *Pseudomonas turukhanskensis* spp. Bacteria culture was spread on agar plate and agar disc containing 2-deoxy-guanosine was added. Migration of cells toward the disc was monitored after 2 h and 24 h*. E. coli* cells were incubated for 24 h, and, then, photo was taken. Note the clearing zones, indicated by arrows, the discs contained 0.1 mM or 1 mM 2DA. *S. marcescens* and *P. turukhanskensis* sp. responded positively at a concentration of 0.1 mM 2DA seen by the clearing zones around the agar discs but not at higher concentration of 1 mM or control discs without 2DA.

To determine whether nucleosides can induce chemotaxis in rhizobacteria as well as in a warm-blooded originating bacterium, we tested the chemoattraction response of *Serratia marcescens*, *Pseudomonas turukhanskensis* spp. and *E. coli* in a plate assay (**Figure 3**). The clearing zones around the agar discs containing 0.1 mM 2DA indicate that all three bacteria are positively chemoattracted to 2DA. It is interesting to note that the clearing zones at a concentration of 1 mM 2DA were smaller than that in agar discs containing 0.1 mM 2DA (data not shown), corresponding to the bell-shaped chemotactic response observed in the capillary assay.

**Figure 4:**
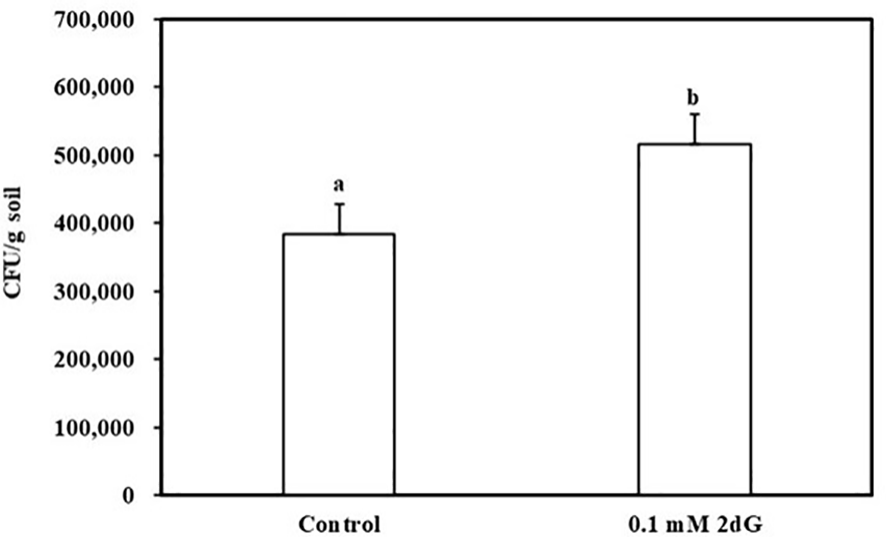
*Bacillus pumilus* b-13 chemotaxis in soil plate assay toward 2-deoxy-guanosine. Bacterial culture was placed in the center of a plate containing moist (20%) dune sand. The outer ring of 0.4% agarose contained 0.1 mM 2-deoxy-guanosine or without (control). After an overnight incubation at 30°C, samples were taken from the perimeter of the soil and colony-forming units (CFU) content was determined. Data are means ± SE, n = 16. Lowercase letters denote statistical significance (α < 0.05) between treatments according to one way-ANOVA, with Tukey HSD.

To test whether nucleosides can diffuse to substantial distances and evoke chemotaxis under conditions as close as possible to natural environments, a soil plate assay was established. The plate contained an outer agar ring with or without 0.1 mM 2DG, and the rest was filled with wet dune sand. *B. pumilus* b-13 was placed at the center of the plate, and, following an overnight incubation, sand samples were taken from the edge of the plate ~ 6-cm radius for CFU counts. The assay shows that *B. pumilus* can traverse 6 cm of wet soil according to 2DG chemical gradient (**Figure 4**). The number of CFU/g sand in 2DG containing plates was significantly higher (5.1 × 10^5^) than that in the control (3.8 × 10^5^). The results show that root secreted nucleosides at biological concentrations presented here can direct bacterial movement toward plants under soil conditions.

**Figure 5:**
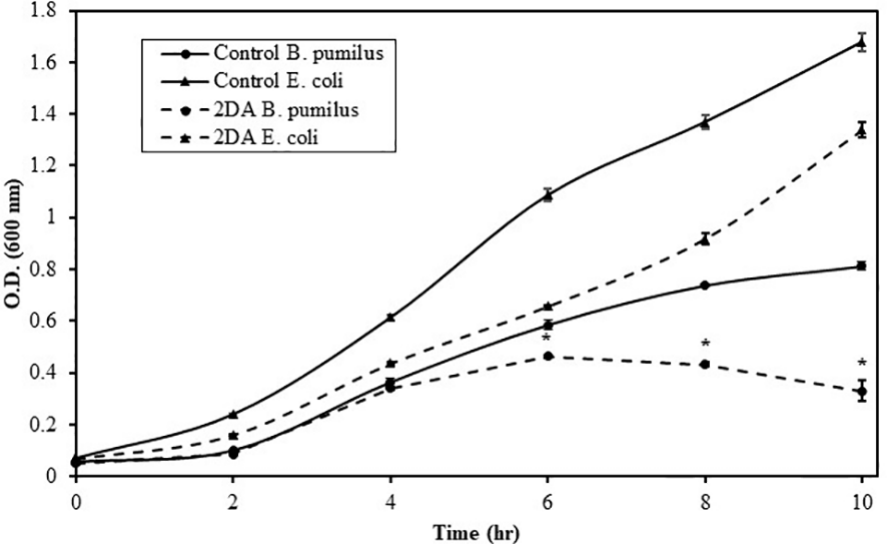
Growth rates of *Bacillus pumilus* (Bp, triangles) and *Escherichia* coli (Ec, filled circles) grown in M9 minimal medium containing glucose or 2-deoxy-adenosine as a carbon source. Bacteria were grown at 34°C, 250 rpm, and O.D._600_ was measured at the indicated intervals. Solid line indicates glucose, and dashed line indicates 2DA. Error bars show the standard error of two technical replicates. Asterisks indicate significant differences (Student’s *t*-test; *P* ≤ 0.05).

Nucleosides are considered poor nutritional source for some Bacilli (Schuch et al., 1999), implying that, in the rhizosphere, they may function as signaling chemoattractant molecules rather than a carbon source. Bacilli comprises a substantial portion of the rhizosphere bacterial community (Satish et al., 2022); therefore, it was important to determine whether 2DA serves as nutrient or signaling molecule. Growing *B. pumilus* and *E. coli* in M9 medium with glucose or 2DA as the carbon source revealed that the growth rate of both bacteria was inhibited (**Figure 5**). *E. coli* uptake nucleosides by several transporter proteins (Patching et al., 2005); however, the average inhibition of growth rate in the presence of 2DA was 30% compared to glucose. *B. pumilus* growth rates were similar on both media up to 4 h, and, after that, the growth rate on 2DA declined to 40% compared to glucose at 10 h. The data indicate that, for *B. pumilus*, 2DA is a signaling chemoattractant and not efficient carbon source to support bacterial growth.

## 4 Discussion

To date, several groups of chemoattractants have been found to be involved in plant–microbe interactions, most of them are nutrients (e.g., amino acids and sugars) and not solely signaling molecules (Feng et al., 2021). This study provides direct evidence that root-secreted nucleosides are an unrecognized class of chemoattractants for both plant beneficial and pathogenic soil-borne bacteria. Because all organisms produce nucleosides, it is conceivable that this mechanism applies to diverse bacterial species and habitats including soils, oceans, rumen, human gut, and many other environments where bacteria can migrate according to a nucleoside chemical gradient (DeLoney-Marino et al., 2003; Crane and Shulgina, 2009; Corriden and Insel, 2012; Ciruela et al., 2021; Pietrangelo, 2021). In accordance, we have found that *E. coli* cells, which inhabit the lower intestine of warm-blooded organisms, formed clearing zones around agar discs containing 1 mM 2DG, in a chemotaxis plate assay (**Figure 3**). Taken together, our data and the above examples strongly support the notion that nucleosides/nucleotides serve as universal chemoattractants among living kingdoms.

The current data suggest that nucleoside signaling is a ubiquitous mechanism for soil bacteria interacting with plants. The following evidence supports this notion: 1) The finding that PGPB bacteria of two separate genera and a pathogenic species exhibit chemoattraction to nucleosides (**Figures 1**–**3**). 2) The identification of nucleosides and deoxynucleosides in root exudates of different plant taxa occupying diverse ecological habitats (**Table 1**). 3) The level of 2DG secreted from *H. sessiliflorum* roots (0.97 ng/7 h, **Table 2**)was >100-fold lower than the nutrient amino acid alanine secreted from wheat roots (89 mg/7 h) (Warren, 2015), further indicating that nucleosides function as specific signaling molecules for some bacteria rather than nutrients. 4) The induction of chemotaxis by very low nucleoside concentrations that probably exist in the soil. In the soil experiment, the 2DG concentration in the outer agar ring was 100 μM (**Figure 4**). The concentration detected in extracts of perlite after growing plants for 44 days was in the range of few micromolar (**Table 2**). Considering the diffusion of 2DG in soil plate assay, it is conceivable that rhizobacteria sensed nucleosides at concentrations of few micromolar, and this was enough to invoke chemotaxis response. 5) The chemotactic response of the rhizobacteria *B. pumilus* to nucleosides in soil (**Figure 3**), indicating that nucleosides can diffuse to substantial distances in soils. Additionally, it was shown that nucleosides are hardly taken up and metabolized by *B. subtilis* (Schuch et al., 1999), and using nucleoside as a sole carbon source decreased the growth rate of *E. coli and B. pumilus* (**Figure 5**). Accordingly, the growth rate of *B. pumilus* in a defined minimal medium was not affected by the omission of nucleosides (Müller et al., 2018).

**Table 2:** Quantification of roots secreted nucleosides.

Plant species	Secreted compound (µg/plant 44 days)							
	Cytidine	2-deoxy-cytidine	Uridine	Guanosine	2-deoxy-guanosine	Thymidine	Adenosine	2-deoxy-adenisine
*Helianthemum sessiliflorum*	N.D.	N.D.	0.155	0.009	0.146	0.008	0.010	0.035
*Arabidopsis thaliana* Col-0	N.D.	N.D.	0.196	N.D.	0.059	0.009	0.025	0.081
*Vigna radiata* (mung beans)	N.D.	N.D.	1.703	0.476	0.589	0.134	0.231	0.957
*Lens culinaris* (lentil)	N.D.	N.D.	0.961	0.164	0.229	0.186	0.298	0.151
*Zea mays* (maize)	N.D.	N.D.	1.610	4.833	5.252	0.530	N.D.	N.D.
*Persea americana* (avocado)	N.D.	N.D.	5.910	2.161	1.854	0.962	N.D.	3.060

The data were normalized by calculating the secreted amount for a plant grown for 44 days. N.D., not detected.

*B. pumilus* chemotaxis response is bell-shaped, unlike the linear response of *B. subtilis* and *X. campestris* (**Figures 1**, **2**). Increased concentrations of secreted nucleosides denote the proximate presence of a host plant, and, for some bacterial species, nucleosides may serve as a regulator switch that changes the flagellar rotary motor mode of action from moving forward to tumbling, consequently enabling the arrested cells to colonize the rhizosphere. This may be attained by the bell-shaped response.

In this work and previous reports, no evidence was found for nucleotide secretion in the tested plant species. A possible explanation for the secretion of nucleosides and not nucleotides is reserving the growth limiting phosphate ions *in planta*. Furthermore, removing the negatively charged phosphate group may facilitate the diffusion of nucleosides in soils that contain positively charged particles.

This is the first report of chemoattraction of a plant pathogen to nucleic acids. Nucleosides elicited a stronger chemoattraction response of *X. campestris* than the amino acids methionine and asparagine (data not shown). *X. campestris* appears to have higher sensitivity toward 2DG compared to 2DA and thymidine (**Figure 2**). Under oxidative stress, which damages DNA, 2DG molecules are formed (Tuteja et al., 2001). Plants attacked by *X. campestris* respond by eliciting oxidative stress, and the formed 2DG may be utilized by the pathogen as a signal denoting a weakened plant.

A prerequisite requirement for nucleobase signaling is the existence of a transport system in plants. Thus far, several nucleobase transporter families have been discovered in plants, but it is not known whether they function in the root excretion system (Girke et al., 2014). As well, the mechanisms of sensing and uptake of nucleosides in both genera *X. campestris* and *B. pumilus* are still unknown, although the existence of a nucleoside uptake protein the NupC transporter in *B. subtilis* was previously reported (Saxild et al., 1996). Our data suggest that bacterial receptors have substantially variable sensitivity to nucleosides and, in the same bacterial species, nucleosides induce chemotrophic response at different threshold levels (**Figures 1**, **2**). *B. pumilus* b-13 is attracted at lower concentrations and with higher motility and sensitivity to nucleosides than the other tested bacteria. In a competition to occupy the rhizosphere, *B. pumilus* will have an advantage over slow moving pathogens. Furthermore, *B. pumilus* possesses an antagonistic effect on the pathogen *X. campestris* and, therefore, seems a promising biocontrol agent (Wulff et al., 2002).

## 5 Conclusion

Future research of nucleoside secretion may reveal chemoattraction response in other PGPB and a potential role in mycorrhiza–bacteria–plant associations as well as in animal–bacteria relations. Investigating the mechanisms and the factors affecting nucleoside secretion is an important and effective tool to establish and support beneficial plant–microbe interactions. Recently, Tang et al. (2022) found that sugarcane/peanut plants grown under an intercropping system secrete substantial amounts of adenine and adenosine, raising the question whether they have potential to promote plant growth by recruiting beneficial rhizobacteria. Our data fully support this notion and the possibility to use nucleosides to improve PGPB recruitment and increase crop yield. It is becoming apparent that PGPB application may reduce plant related biotic and abiotic stresses, increase production, and amend the environmental damage created by over-fertilization (Compant et al., 2019). Uncovering the mechanisms involved in the assembly and establishment of microbial communities in the rhizosphere is crucial to improve agricultural practices. The data presented here contribute to the understanding of how plants organize the biome community in the rhizosphere.

## Data availability statement

The original contributions presented in the study are included in the article/supplementary material, further inquiries can be directed to the corresponding author/s.

## Author contributions

GK: Writing – original draft, Writing – review & editing, Investigation. GY: Investigation, Writing – review & editing. LS: Investigation, Writing – review & editing, Methodology. ZA: Investigation, Writing – review & editing. ZB: Conceptualization, Writing – review & editing. SB-S: Conceptualization, Supervision, Writing – review & editing. VK-Z: Conceptualization, Supervision, Writing – review & editing. YS: Conceptualization, Funding acquisition, Supervision, Writing – original draft, Writing – review & editing.
